# Maternal Body Mass Index and Risk of Congenital Heart Defects in Infants: A Dose-Response Meta-Analysis

**DOI:** 10.1155/2019/1315796

**Published:** 2019-07-07

**Authors:** Xuezhen Liu, Guoyong Ding, Weili Yang, Xia Feng, Yuejin Li, Huamin Liu, Qianqian Zhang, Long Ji, Dong Li

**Affiliations:** School of Public Health, Shandong First Medical University & Shandong Academy of Medical Sciences, Taian, China

## Abstract

**Objective:**

The exact shape of the dose-response relationship between maternal body mass index (BMI) and the risk of congenital heart defects (CHDs) in infants has not been clearly defined yet. This study aims to further clarify the relationship between maternal obesity and the risk of CHDs in infants by an overall and dose-response meta-analysis.

**Methods:**

PubMed, Embase, and Web of Science databases were searched to identify all related studies. The studies were limited to human cohort or case-control studies in English language. Random-effect models and dose-response meta-analysis were used to synthesize the results. Heterogeneity, subgroup analysis, sensitivity analysis, and publication bias were also assessed.

**Results:**

Nineteen studies with 2,416,546 participants were included in our meta-analysis. Compared with the mothers with normal weight, the pooled relative risks (RRs) of infants with CHDs were 1.08 (95% CI=1.03-1.13) in overweight and 1.23 (95% CI=1.17-1.29) in obese mothers. According to the findings from the linear meta-analysis, we observed an increased risk of infants with CHDs (RR=1.07, 95% CI=1.06-1.08) for each 5 kg/m^2^ increase in maternal BMI. A nonlinear relationship between maternal BMI and risk of infants with CHDs was also found (*p*=0.012).

**Conclusion:**

The results from our meta-analysis indicate that increased maternal BMI is related to increased risk of CHDs in infants.

## 1. Introduction

Congenital heart defects (CHDs), which account for nearly one-third of all major congenital anomalies, are the most common birth defects in newborns [1]. As the serious medical problem, CHDs play a very important role in the death of newborns and infants [2, 3]. Epidemiological investigations have documented that the prevalence of CHDs in infants is differentiated in regions with an estimated prevalence of 4 to 10 cases per 1,000 births [4]. It is reported that the number of infants with CHDs worldwide has notably increased with more than one million annually [5]. Identifying modifiable risk factors of infants with CHDs remains important for public health and clinical medicine. The exact etiologies of CHDs are complex, several causes such as genetic factors [6–8], physical and chemical factors [9–12], infection during pregnancy [13, 14], medication during pregnancy [15, 16], and mental health status or diseases during pregnancy [17–20] have been identified. However, there are still some potential risk factors that have not been fully confirmed, such as maternal obesity.

Obesity has become a major public health problem that challenges both developed and developing countries [21–23]. Data from epidemiological research showed that women of childbearing age accounted for a large proportion of obese population [24]. The association between maternal obesity and CHDs in infants has been widely reported, but the results are not consistent. For example, one cohort study by Persson et al. suggested that maternal obesity significantly increased the risk of CHDs in infants, and Brite et al. also confirmed the positive association in their study [25, 26]. However, Rankin et al. and Gharderian et al. demonstrated that there was no significant association between increased maternal BMI and increased CHDs risk in offspring [27, 28]. Therefore, the evidence from these observational studies has been inconsistent.

As the dose-response meta-analysis is a reliable quantitative measure of causality, in our study, we conducted a dose-response meta-analysis on maternal BMI and the risk of CHDs in infants by synthesizing the results of published original studies. Our aim was to clearly delineate the shape of the dose-response relationship between maternal BMI and CHDs in infants and to examine the possibility of the nonlinear relationships.

## 2. Materials and Methods

### 2.1. Search Strategy

We systematically searched PubMed, Embase, and Web of Science databases to April 31, 2018, for studies on the relationship between maternal BMI and infants with CHDs. The following search strategy was used: (congenital heart defects OR congenital malformations OR birth defects OR CHD OR CHDs) AND (overweight OR obesity OR body mass index OR BMI). Additional possible relevant publications were identified by reviewing the references lists of retrieved articles and published meta-analysis. The searched studies were strictly limited to human cohort studies or case-control studies in English language.

### 2.2. Study Selection

Studies satisfying the following criteria were included in our meta-analysis: (1) cohort or case-control study design; (2) having clear BMI categories of prepregnancy or early pregnancy; (3) CHDs or one of the CHD subtypes as outcome; (4) relative risk (RR) or odds ratio (OR) with 95% confidence intervals (CIs) available or having sufficient published data to calculate them. In addition, the study for dose-response analysis had to report the estimates of at least three BMI classifications. The Newcastle-Ottawa Scale in which the star system ranges from 0 to 9 was used to assess the methodological quality of studies, and a study awarded seven or more stars was considered high-quality and was included in the meta-analysis [29, 30]. When multiple studies reported the duplicated data, only the latest one with completed data was included.

### 2.3. Data Extraction

Data were extracted by 2 independent investigators (X.L. and W.Y.), and any disagreement was resolved through consensus from another author (L.J.). The following variables were collected from each publication: first author's name, publication year, study location, study period, study sample size, number of cases, study design, BMI category and the corresponding risk estimate, confounding factors adjusted in multivariable analysis, and study conclusion. Considering that the rate ratio, risk ratio, and hazard ratio can be used as a valid estimate of the relative risk and the meaning of the odds ratio is similar to the relative risk, then we used the RRs to report the results for convenience. In order to reduce the impact of covariates, the adjusted RRs in multivariate analysis were preferentially extracted.

The average BMI corresponding to each classified RR was calculated by the midpoint of the upper and lower boundary of each category. In the case where the highest category or the lowest category was the open interval, we assumed that they had the same amplitudes as the adjacent category [31]. When a study provided only total number of cases and person-years, the distribution of cases and person-years were estimated through the method described by Aune et al. [32].

### 2.4. Statistical Analysis

We conducted separate meta-analysis to calculate the pooled RRs and 95% CIs for overweight and obese mothers versus normal-weight mothers. For the category of BMI, we used the classification standard of WHO (underweight, <18.5 kg/m^2^; normal weight, 18.5-24.9 kg/m^2^; overweight, 25.0-29.9 kg/m^2^; obesity, ≥30.0 kg/m^2^) [33, 34]. The logarithmic transformations for the RRs and the corresponding standard errors extracted from studies were performed to make the variances stabilized and the distributions normalized. A random-effects model was used to combine the estimates [34]. The random-effects model was chosen a priori because it was considered as more conservative than the fixed-effects model, as it accounted for both within- and between-study heterogeneity [35]. The* I*^2^ statistic and the* Q*-test were used to assess the heterogeneity across studies, and* I*^2^ values of 0, 25%, 50%, and 75% were considered indicative of no, low, moderate, and high heterogeneity, respectively [36]. Considering that the relationship between maternal obesity and CHDs in infants may be affected by study-specific factors (e.g., study design, study location, study sample size, maternal age, smoking, and education), subgroup analyses were separately conducted based on these possible confounders.

A two-stage random-effect dose-response meta-analysis, which required the variables of cases, person-years, mean level of BMI, and the corresponding RR in each category, was used to depict the trend from the relevant log-RRs estimated across BMI categories, considering the heterogeneity between studies[37]. In the first stage, a generalized least squares regression was used to estimate the restricted cubic spline model with three knots at the 10th, 50th, and 90th percentiles of the distribution, considering the correlation within each set of the published RRs. Then, the estimates value for each study calculated in the previous step was merged to carry out the dose-response relationship between maternal BMI and the risk of infants with CHDs. The null hypothesis that the second spline coefficient is equal to zero was tested to calculate the* p* value for nonlinearity [38].

In addition, we conducted sensitivity analysis, in which one study involved in the meta-analysis was eliminated at a time and the rest pooled to evaluate the stability of our results [39]. Evidence of publication bias was appraised through funnel plots and Egger's regression tests [40]. All statistical analyses were performed by Stata 12.0 (Stata Corporation, College Station, TX). A* p* value less than 0.05 was considered statistically significant, except for the Q-test (*p*<0.10) because of the low power of the test.

## 3. Results

### 3.1. Literature Search and Study Characteristics

**Figure 1:**
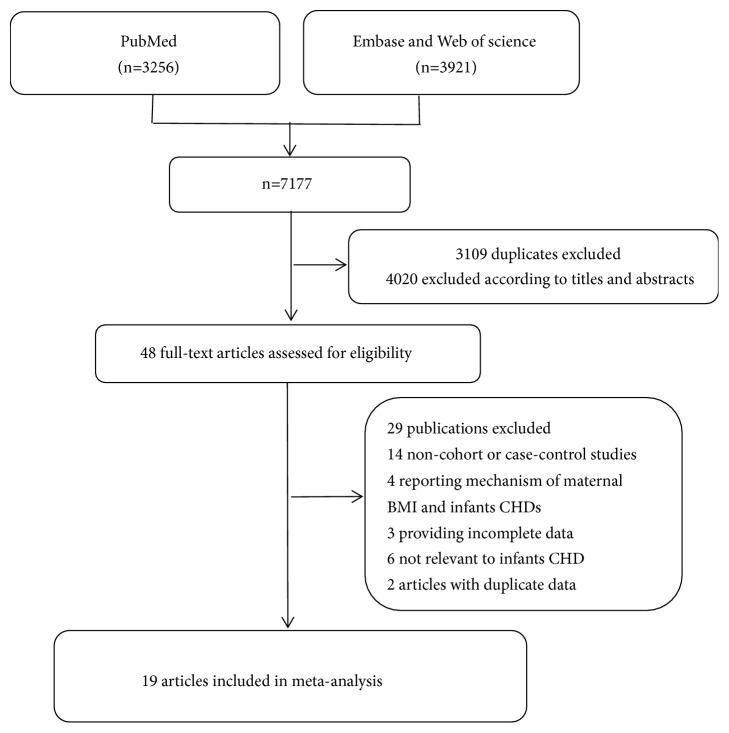
Flowchart of the selection of studies for inclusion in this meta-analysis.

Our meta-analysis included 6 cohort studies [25–27, 41–43] and 13 case-control studies [28, 44–55], which involved 57,172 cases and 2,416,546 participants (Figure 1). Among these studies, 12 were conducted in the North America [26, 28, 41, 43–47, 50, 51, 53, 55], 4 in Europe [25, 27, 42, 54], one in Oceania [48], and 2 in Asia [49, 52]. A total of 10 studies had less than 10,000 participants [28, 44, 48–55] while nine studies had more than 10,000 participants [25–27, 41–43, 45–47]. Eight studies controlled for maternal age [25–27, 46–48, 51, 55] and 7 studies controlled for maternal smoking [25–27, 46, 47, 51, 55]. For the factor of maternal education, it was adjusted in 6 studies [25, 46–48, 51, 55]. Of the included studies, 9 reported that maternal obesity significantly increased the risk of CHDs in infants [25, 26, 42, 44–47, 51, 53], and 10 reported that there was no significant association between increased maternal BMI and increased CHDs risk in offspring [27, 28, 41, 43, 48–50, 52, 54, 55]. The general characteristics of the included studies were shown in Table 1.

### 3.2. Abnormal Maternal BMI and Infants with CHDs

**Figure 2:**
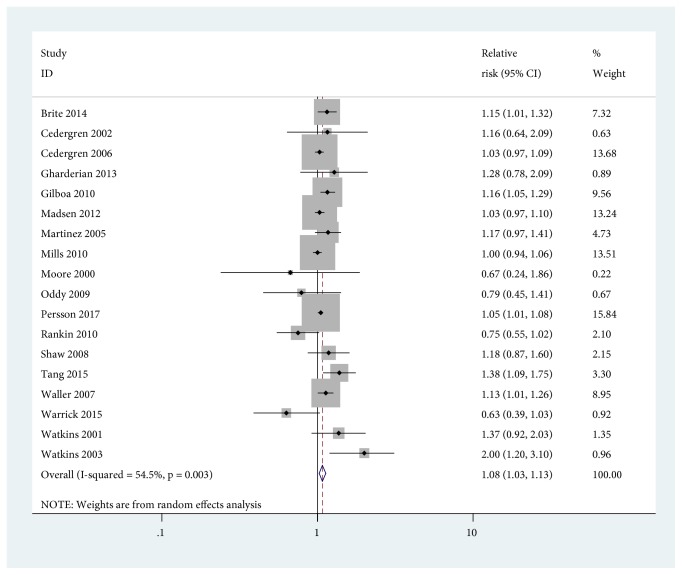
*Forest plot of RRs of maternal overweight versus maternal normal weight for BMI with CHDs risk in infants*. RR, relative risk; CI, confidence interval; BMI, body mass index.

Compared with maternal normal weight, the pooled RR of CHDs in infants was 1.08 (95% CI=1.03-1.13) for maternal overweight and some evidence of heterogeneity across studies was found with* I*^2^=54.5% (Figure 2). Subgroup analysis suggested that the pooled association of CHDs in infants among overweight mothers was significantly higher in studies with less than 10,000 participants (RR=1.21, 95% CI=1.10-1.34) than that in studies with more than 10,000 participants (RR=1.04, 95% CI=1.00-1.09). In addition, the corresponding* I*^2^ statistics were 16.1% and 54.9%, respectively, which indicated that the heterogeneity was derived from studies with sample sizes more than 10,000. Meanwhile, the pooled RR and the* I*^2^ statistic for studies conducted in the United States were 1.12 (95% CI=1.04-1.21) and 62.7%, while the pooled RR and the* I*^2^ statistic for studies conducted outside the United States were 1.04 (95% CI=0.99-1.10) and 30.4%, which demonstrated that American studies resulted in the heterogeneity (Table 2).

**Figure 3:**
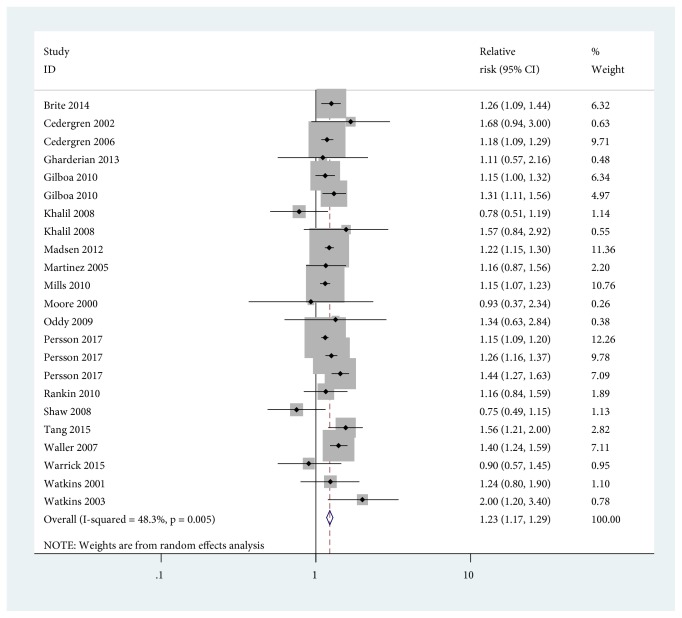
Forest plot of RRs of maternal obesity* versus* maternal normal weight for BMI with CHDs risk in infants. RR, relative risk; CI, confidence interval; BMI, body mass index.

Using mothers with normal BMI as the reference category, we found that maternal obesity increased the risk of CHDs in infants (RR=1.23, 95% CI=1.17-1.29). No evidence of high heterogeneity was found for the category of obesity (*I*^2^ = 48.3%) (Figure 3). When stratified by study design, the pooled RR of infants with CHDs among obese mothers was 1.22 (95% CI=1.15-1.31) compared with mothers with normal weight in cohort studies, and the pooled RR among obese mothers was 1.24 (95% CI=1.15-1.33) compared with mothers with normal weight in case-control studies. It was noted that the effect differences were not observed for study design, study location, study sample sizes, and other adjustment factors (e.g., maternal age, maternal smoking, and maternal education) (Table 2).

### 3.3. Dose-Response Meta-Analysis

**Figure 4:**
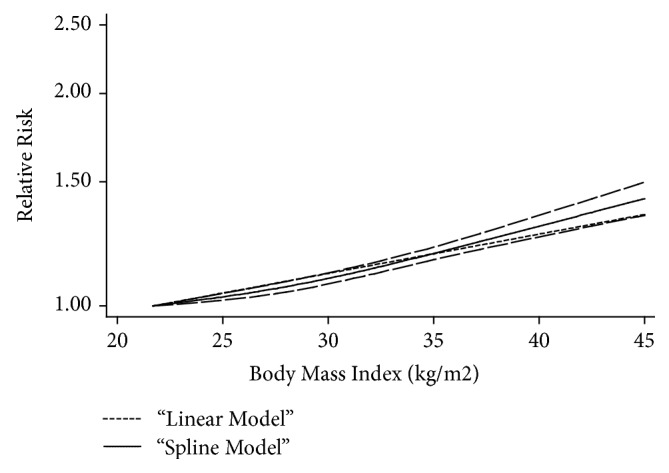
*The dose-response analysis between maternal BMI and CHDs risk in infants with restricted cubic splines in a multivariate random-effects dose-response model*. The solid line and the long dash line represent the estimated RR and its 95% CI. Short dash line represents the linear relationship (per 5 kg/m^2^ increment). RR, relative risk; CI, confidence interval; BMI, body mass index.

**Figure 5:**
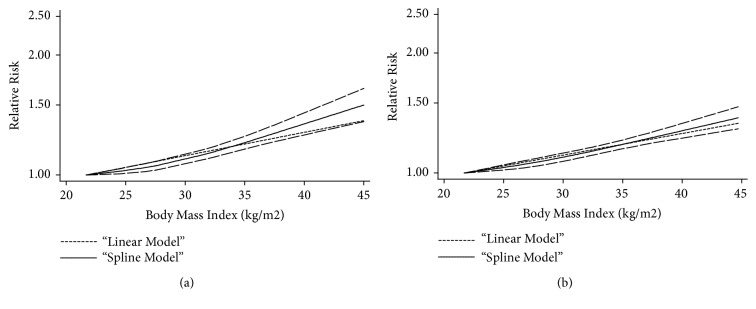
*The dose-response analysis between maternal BMI and CHDs risk in infants by adjustment of study design*. (a) Cohort studies; (b) case-control studies. The solid line and the long dash represented RR and its 95% CI. Short dash line represents the linear relationship (per 5 kg/m^2^ increment). RR, relative risk, CI, confidence interval; BMI, body mass index.

All of the above 19 studies were included in the dose-response meta-analysis of maternal BMI and risk of infants with CHDs. As shown in Figure 4, an increased risk of CHDs in infants (RR=1.07, 95% CI=1.06-1.08) for each 5 kg/m^2^ increase in maternal BMI was shown in this meta-analysis. When stratified by study design, it was found that the risk of infants with CHDs increased by 7% for every 5 kg/m^2^ increase of maternal BMI, in both cohort studies (RR=1.07, 95% CI=1.06-1.08) and case-control studies (RR=1.07, 95% CI=1.05-1.09) (Figure 5).

As shown in Figure 4, it was found that there was a nonlinear relationship between maternal BMI and risk of CHDs in infants (*p*=0.012). Compared with BMI=22.05 kg/m^2^, the pooled RRs (95% CIs) of infants with CHDs were 1.03 (95% CI=1.02-1.04), 1.08 (95% CI=1.06-1.10), 1.18 (95% CI=1.16-1.21), 1.36 (95% CI=1.30-1.42), and 1.42 (95% CI=1.34-1.50) for BMI=25, 30, 35, 40, and 45 kg/m^2^, respectively. The evidence of significant nonlinear relationship was also observed in cohort studies (*p*=0.015) when adjusting the factor of study design. At the points of BMI=25, 30, 35, 40, and 45 kg/m^2^, the corresponding RRs (95% CIs) for cohort studies were 1.02 (95% CI=1.01-1.04), 1.13 (95% CI=1.10-1.16), 1.21 (95% CI=1.16-1.25), 1.42 (95% CI=1.31-1.54), and 1.50 (95% CI=1.36-1.65), respectively (Figure 5).

### 3.4. Publication Bias

**Figure 6:**
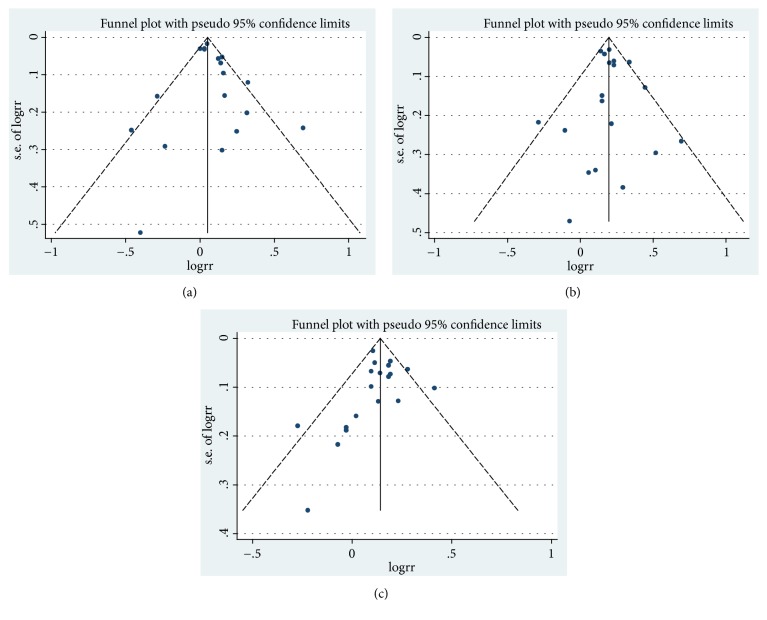
*Funnel plot corresponding to the random-effects meta-analysis of the relationship between* (a) maternal overweight and infants CHDs risk (*p*=0.346, by Egger's test); (b) maternal obesity and infants CHDs risk (*p*=0.744, by Egger's test); (c) funnel plot corresponding to the dose-response meta-analysis of the relationship between maternal BMI and infants CHDs risk (*p*=0.605, by Egger's test). BMI, body mass index; CHD, congenital heart defects.

Egger's regression tests showed no evidence of publication bias in the literature about maternal BMI and risk of infants with CHDs in maternal overweight group (*p*=0.346), maternal obesity group (*p*=0.744), and dose-response group (*p*=0.605) (Figure 6).

### 3.5. Sensitivity Analysis

In a sensitivity analysis in which one study at a time was eliminated and the remaining analyzed, the pooled RRs of infants with CHDs ranged from 1.07 to 1.09 for maternal overweight group, from 1.21 to 1.24 for maternal obesity group, and from 1.15 to 1.17 for dose-response analysis group separately, which demonstrated that the pooled estimates were steady and not affected by a single study.

## 4. Discussion

In the present meta-analysis, we discovered an increase of 8% risk of infants with CHDs in maternal overweight group and an increase of 23% risk in maternal obesity group compared with the mothers with normal weight. Subgroup analysis by study design showed that the significant association between maternal overweight and increased risk of infants with CHDs existed only in case-control studies, while the significant association between maternal obese and increased risk of infants with CHDs existed in both cohort studies and case-control studies. Dose-response meta-analysis showed that each 5 kg/m^2^ increase of maternal BMI is accompanied by a 7% increment of risk of infants with CHDs, and a significantly nonlinear relationship between maternal BMI and infants with CHDs risk was observed (*p=*0.012). When stratified by study design, the pooled RR of infants with CHDs increased by 7% per 5 kg/m^2^ increase of maternal BMI, for both cohort and case-control studies. The evidence of significant nonlinear relationship between maternal BMI and risk of infants with CHDs was also found in cohort studies (*p=*0.015).

Our findings are similar to meta-analysis by Cai et al., who examined the association between maternal BMI and CHDs in offspring and reported a similar summary for overweight and obese individuals [56]. However, that meta-analysis only included 14 studies and the possibility of nonlinear association between maternal BMI and infants with CHDs was not reported. In another meta-analysis, a slightly lower significant association between maternal overweight and increased CHDs risk in infants and a significant association between maternal obesity and CHDs in their offspring were observed [57]. Nevertheless, the dose-response relationship was also not examined in their meta-analysis. Our results, based on 20 studies, were generally in line with the results of previous meta-analysis [56, 57]. Moreover, the statistically nonlinear dose-response relationship between maternal BMI increase and risk of infants with CHDs was also found in our study. In addition, we conducted subgroup analysis through possible confounding factors such as study design, study sample sizes and adjustment factors, which made our result more abundant.

Maternal obesity might be associated with increased risk of infants with CHDs through several mechanisms. Data from epidemiology research suggest that folate, glutathione, and homocysteine metabolism related genetic variants in maternal and fetal may have great impact on the heart development [57]. It had been reported that obesity mothers who carried mutant genotype AC for glutamate-cysteine ligase, catalytic subunit (*GCLC*) gene (rs6458939) significantly increased the risk of conotruncal defects (CTDs) in infants, compared with those obesity mothers who carried the CC genotype [58]. Another possible mechanism is that maternal metabolic environment plays an important role in fetal developments [59]. Decreased intake of folate and glutathione and increased intake of homocysteine caused by maternal obesity may lead to abnormal in utero environment, which contribute to the onset and development of impaired fetal developments [60–64]. Additionally, some animal studies have reported on possible ways of maternal obesity-mediated offspring CHDs. Firstly, through changing the signal path, Wang et al. reported that diabetes-induced heart defects may be affected by apoptosis signal-regulating kinase 1 (ASK1), which can be attributed to the activation of ASK1 on c-Jun NH2-terminal kinase 1/2 (JNK 1/2)-endoplasmic reticulum (ER) stress pathway, inhibition of ASK1 on cell cycle progression, and mediation of ASK1 on teratogenicity of diabetes [65]. Another study demonstrated that maternal obesity in sheep pregnancy can alter the JNK-IRS-1 signaling cascades and cardiac function in the fetal heart [66]. Huang et al. indicated that maternal obesity results in greater fetal heart connective tissue accumulation associated with an upregulated TGF-*β*/p38 signaling pathway at late gestation, and such changes may negatively impact offspring heart function [67]. Secondly, it was reported that maternal obesity may impair fetal cardiomyocyte contractility and affect cardiac development by altering intracellular Ca^2+^ treatment, overloading fetal Ca^2+^, and abnormal myofibrillar proteins [68]. Thirdly, maternal obesity significantly enhances TLR4, IL-1a, IL-1b, and IL-6 expression, promotes phosphorylation of I-*κ*B, decreases cytoplasmic NF-*κ*B levels, and increases neutrophil and monocyte infiltration, eventually leading to inflammation in the fetal heart and altering fetal cardiac morphometry [69]. Furthermore, a mini-review by Dong et al. reported that lipotoxicity resulting from maternal obesity is capable of activating a number of stress signaling cascades including proinflammatory cytokines and oxidative stress to exacerbate cardiovascular complications [70].

The present meta-analysis had some advantages. Firstly, more relevant original studies and a large number of participants and cases were included, which significantly improved the statistical power of the analysis. Meanwhile, we conducted a quality assessment of eligible studies using the Newcastle-Ottawa Scale, and the included studies can be considered as high-quality because they all awarded seven or more stars. Secondly, the dose-response meta-analysis was performed, and the possibility of nonlinear relationship was evaluated in our study, which made the association between maternal BMI and the risk of CHDs in infants better described. In spite of these strengths, the interpretation of the results in our meta-analysis may be affected by several potential limitations. First, most studies included in our meta-analysis were case-control studies and it is reported that the information bias might be more prone to occur in case-control studies than cohort studies. Then, some confounding factors (e.g., maternal age, maternal smoking, and maternal education) only were adjusted in very few included studies, which may lead to an overestimation of the true association between maternal obesity and risk of CHDs in offspring. Finally, it is impossible to completely exclude the potential publication bias because some studies with invalid results tend not to be published.

## 5. Conclusion

In conclusion, our overall and dose-response meta-analysis indicate that increased maternal BMI is related to increased risk of CHDs in infants. The measures of maternal weight control before they plan to conceive are necessary to decrease the risk of CHDs in infants. The findings from our meta-analysis need to be confirmed in well-designed intervention studies in the future.

## Figures and Tables

**Table 1:** Characteristics of included studies.

Author (year)	Country	Study period	Study size no	No of cases	Study design	BMI (kg/m^2^)	RR (95%CI)	Adjustment factors	Study conclusion	NOS
Persson, 2017	Sweden	2001-2014	1,243,957	20,074	Cohort study	<18.5 18.5-24.9 25.0-29.9 30.0-34.9 35.0-39.9 ≥40.0	0.99(0.90-1.09) 1.00 1.05(1.01-1.08) 1.15(1.09-1.20) 1.26(1.16-1.37) 1.44(1.27-1.63)	Maternal age, height, parity, early pregnancy, smoking status, education level, maternal country of birth, family situation, sex of offspring	Risks of infants CHDs progressively increased with increasing severity of maternal overweight and obesity.	8

Warrick, 2015	The United States	2005-2011	18,226	117	Cohort study	<18.5 18.5-24.9 25.0-29.9 ≥30.0	0.61(0.22-1.67) 1.00 0.63(0.39-1.03) 0.90(0.57-1.45)	NA	No significant differences in maternal obesity between mothers with and without CHDs infants were shown.	7

Brite, 2014	The United States	2002-2008	121,815	1,388	Cohort study	<18.5 18.5-24.9 25.0-29.9 ≥30.0	1.08(0.85-1.38) 1.00 1.15(1.01-1.32) 1.26(1.09-1.44)	Site, maternal age, race, insurance, maternal smoking	Increasing maternal weight class was associated with increased risk for CHDs in infants.	7

Rankin, 2010	England	2003-2005	30,703	270	Cohort study	<18.5 18.5-24.9 25.0-29.9 ≥30.0	1.55(0.90-2.66) 1.00 0.75(0.55-1.02) 1.16(0.84-1.59)	Maternal age, ethnicity, pre-gestational diabetes, cigarette smoking status, index of multiple deprivation.	No significant associations were found between maternal BMI and infants CHDs risk.	7

Cedergren, 2006	Sweden	1992-2001	770,355	6,346	Cohort study	<20.0 20.0-24.9 25.0-29.9 ≥30.0	0.97(0.89-1.05) 1.00 1.03(0.97-1.09) 1.18(1.09-1.29)	NA	Maternal obesity was more common in pregnancies with infants affected by CHDs.	7

Moore, 2000	The United States	1984-1987	22,951	60	Cohort study	<25.0 25.0-27.9 ≥28.0	1.00 0.67(0.24-1.86) 0.93(0.37-2.34)	NA	There was no evidence of an excess risk of CHDs in infants among the obese women.	7

Tang, 2015	The United States	1997-2008	2,147	553	Case-control study	<18.5 18.5-24.9 25.0-29.9 ≥30.0	0.64(0.35-1.15) 1.00 1.38(1.09-1.75) 1.56(1.21-2.00)	NA	The risk of CHDs was closely related to maternal obesity.	7

Gharderian, 2013	The United States	2011-2012	322	164	Case-control study	<18.5 18.5-24.9 25.0-29.9 ≥30.0	0.85(0.32-2.27) 1.00 1.28(0.78-2.09) 1.11(0.57-2.16)	NA	There might not be a relation between maternal BMI and having a child with CHDs.	7

Madsen, 2012	The United States	1992-2007	107,901	7,547	Case-control study	<18.5 18.5-24.9 25.0-29.9 ≥30.0	1.02(0.91-1.15) 1.00 1.03(0.97-1.10) 1.22(1.15-1.30)	Gestational diabetes	The significant association between infants CHDs and maternal obesity was confirmed.	8

Gilboa, 2010	The United States	1998-2003	12,113	6,440	Case-control study	<18.5 18.5-24.9 25.0-29.9 30.0-34.9 ≥35.0	0.96(0.80-1.16) 1.00 1.16(1.05-1.29) 1.15(1.00-1.32) 1.31(1.11-1.56)	Maternal age, race-ethnicity, education, hypertension, parity, smoking, folic acid supplement use	Mothers of CHDs infants were more likely than mothers of control infants to be overweight, moderately obese or severely obese.	7

Mills, 2010	The United States	1993-2003	63,696	7,392	Case-control study	<19.0 19.0-24.0 25.0-29.0 ≥30.0	1.00(0.91-1.10) 1.00 1.00(0.94-1.06) 1.15(1.07-1.23)	Maternal age, education, race, smoking, and payment method for health care.	Obese, but not overweight, women are at significantly increased risk of bearing children with CHDs.	8

Oddy, 2009	Australia	1997-2000	529	111	Case-control study	<20.0 20.0-24.9 25.0-29.9 ≥30.0	0.74(0.40-1.36) 1.00 0.79(0.45-1.41) 1.34(0.63-2.84)	Marital status, maternal age, maternal education and periconceptional folic acid supplementation	No significant associations were found between maternal BMI and infants CHDs risk.	8

Khalil, 2008	Saudi Arabia	1998-2005	428	214	Case-control study	19.0-25.0 30.0-34.9 ≥35.0	1.00 0.78(0.51-1.19) 1.57(0.84-2.92)	NA	No association was found between maternal weight and isolated CHDs in the offspring.	7

Shaw, 2008	The United States	1999-2004	1578	278	Case-control study	<18.5. 20.0-24.9 25.0-29.9 ≥30.0	0.84(0.46-1.56) 1.00 1.18(0.87-1.60) 0.75(0.49-1.15)	NA	The association between maternal BMI and CHDs in infants was not significant.	7

Waller, 2007	The United States	1997-2002	8032	4128	Case-control study	<18.5. 20.0-24.9 25.0-29.9 ≥30.0	1.12(0.93-1.36) 1.00 1.13(1.01-1.26) 1.40(1.24-1.59)	Maternal age, ethnicity, education, parity, smoking in the month prior to conception, and supplemental folic acid intake	Obesity or overweight women had a modest increase in the risk of infants CHDs.	8

Martinez, 2005	Spain	1976-2001	6973	813	Case-control study	≤20.9 21.0-24.9 25.0-29.9 ≥30.0	1.00(0.83-1.20) 1.00 1.17(0.97-1.41) 1.16(0.87-1.56)	NA	Maternal overweight or obesity did not increase the risk of CHDs in infants.	7

Watkins, 2003	The United States	1993-1997	525	195	Case-control study	<18.5. 20.0-24.9 25.0-29.9 ≥30.0	1.70(0.90-3.10) 1.00 2.00(1.20-3.10) 2.00(1.20-3.40)	NA	The significant association between infants CHDs and maternal obesity was confirmed.	7

Cedergren 2002	Sweden	1982-1996	677	231	Case-control study	<19.8. 19.8-25.9 26.0-28.9 ≥29.0	1.46(0.97-2.21) 1.00 1.16(0.64-2.09) 1.68(0.94-3.00)	NA	The associations between maternal BMI and infants CHDs risk was not confirmed.	7

Watkins, 2001	The United States	1982-1983	3618	851	Case-control study	<16.5. 16.5-19.8 19.9-22.7 22.8-26.0 26.1-29.0 >29.0	0.78(0.55-1.11) 0.97(0.81-1.17) 1.00 0.84(0.67-1.06) 1.37(0.92-2.03) 1.24(0.80-1.90)	Race, birth period, age, education, alcohol use, smoking, chronic illness, and vitamin use	There might not be a relation between maternal BMI and having a child with CHDs.	8

BMI, body mass index; RR, relative risk; CI, confidence interval; NA, not available; NOS, Newcastle-Ottawa Scale.

**Table 2:** Subgroup analysis of maternal BMI and CHDs risk in infants.

*Study*	Overweight			Obesity		
	No.of studies	RR (95%CI)	*I* ^2^(%)	No.of studies	RR (95%CI)	*I* ^2^(%)
All studies	18	1.08(1.03-1.13)	54.5	19	1.23(1.17-1.29)	48.3
*Study design*						
Cohort	6	1.03(0.96-1.11)	56.9	6	1.22(1.15-1.31)	53.2
Case-control	12	1.13(1.05-1.21)	56.1	13	1.24(1.15-1.33)	48.7
*Study location*						
The United States	12	1.12(1.04-1.21)	62.7	12	1.24(1.15-1.32)	48.3
Not the United States	6	1.04(0.99-1.10)	30.4	7	1.22(1.14-1.32)	52.1
*Sample sizes*						
Less than 10000	9	1.21(1.10-1.34)	16.1	10	1.27(1.08-1.49)	49.3
More than 10000	9	1.04(1.00-1.09)	54.9	9	1.21(1.16-1.26)	38.8
*Adjustment factors*						
*Maternal age*						
Yes	8	1.07(1.01-1.14)	58.3	8	1.24(1.17-1.31)	54.1
No	10	1.11(1.01-1.22)	56.2	11	1.20(1.08-1.33)	47.0
*Maternal smoking*						
Yes	7	1.07(1.01-1.14)	62.1	7	1.24(1.17-1.31)	58.5
No	11	1.10(1.00-1.21)	53.5	12	1.20(1.09-1.33)	42.4
*Maternal education*						
Yes	6	1.07(1.01-1.13)	52.4	6	1.24(1.17-1.33)	62.5
No	12	1.09(1.00-1.19)	59.1	13	1.21(1.11-1.31)	38.6

BMI, body mass index; CHDs, congenital heart defects; RR, relative risk; CI, confidence interval.

## Supplementary Materials

Supplementary Materials Scheme: the scheme of the meta-analysis. Supplementary Figure: figures of sensitivity analysis. Supplementary Table: the checklist of PRISMA Statement.
