# Supplementary material for: Maternal Body Mass Index and Risk of Congenital Heart Defects in Infants: A Dose-Response Meta-Analysis
Source: Biomed Res Int. 2019 Jul 7;2019:1315796. doi: 10.1155/2019/1315796 (PMC6642764; doi:10.1155/2019/1315796)
Supplement: Supplementary Materials — Scheme: the scheme of the meta-analysis. Supplementary Figure: figures of sensitivity analysis. Supplementary Table: the checklist of PRISMA Statement. [file 1315796.f1.pdf]

Supplementary material

Scheme:

Specific research question: Does maternal obesity increase the risk of congenital heart defects (CHDs) in infants?

Research population: Obese or overweight mother, congenital heart defects infants

Selection criteria for original research: Studies satisfying the following criteria were included in our meta-analysis: 1) cohort or case-control study design; 2) having clear BMI categories of pre-pregnancy or early pregnancy; 3) CHDs or one of the CHD subtypes as the outcome; 4) relative risk (RR) or odds ratio (OR) with 95% confidence intervals (CIs) available or had sufficient published data to calculate them. In addition, the study for dose-response analysis had to report the estimates of at least three BMI classifications. When multiple studies reported the duplicated data, only the most recent one with completed data was included.

Exposure and outcome: The classification criteria for BMI as exposure are as follows: underweight, <18.50; normal weight, 18.50-24.90; overweight, 25.0-29.90; obesity, ≥30.00. CHDs or one of the CHD subtypes as the outcome.

Determining search strategy: The following search strategy was used: (congenital heart defects OR congenital malformations OR birth defects OR CHD OR CHDs) AND (overweight OR obesity OR body mass index OR BMI). Additional possible relevant publications were identified by reviewing the references lists of retrieved articles and published meta-analysis. The search was strictly limited to human cohort studies or case-control studies in English language.

Evaluation of research quality: The Newcastle-Ottawa Scale in which the star system ranges from 0 to 9 was used to assess the methodological quality of studies.

Data extraction: The following variables were collected from each publication: first author's name, publication year, study location, study period, study sample size, number of cases, study design, BMI category and the corresponding risk estimate, and confounding factors adjusted in multivariate analysis. In order to reduce the impact of covariates, the adjusted RRs in multivariate analysis were preferentially extracted.

Statistical Analysis: The I^2 statistic and the Q-test were used to assess the heterogeneity across studies. A random-effects model was used to combine the estimates. Subgroup analysis was separately conducted based on possible confounders. A two-stage random-effect dose-response meta-analysis was used to depict the trend from the relevant logRRs estimated across BMI categories. In addition, we conducted a sensitivity analysis, in which one study involved in the meta-analysis was eliminated at a time and the rest pooled to evaluate the stability of our results. Evidence of publication bias was estimated by funnel plots and Egger's regression test.

Paper writing: Paper was written by Xuezhen Liu.

Supplementary Figure

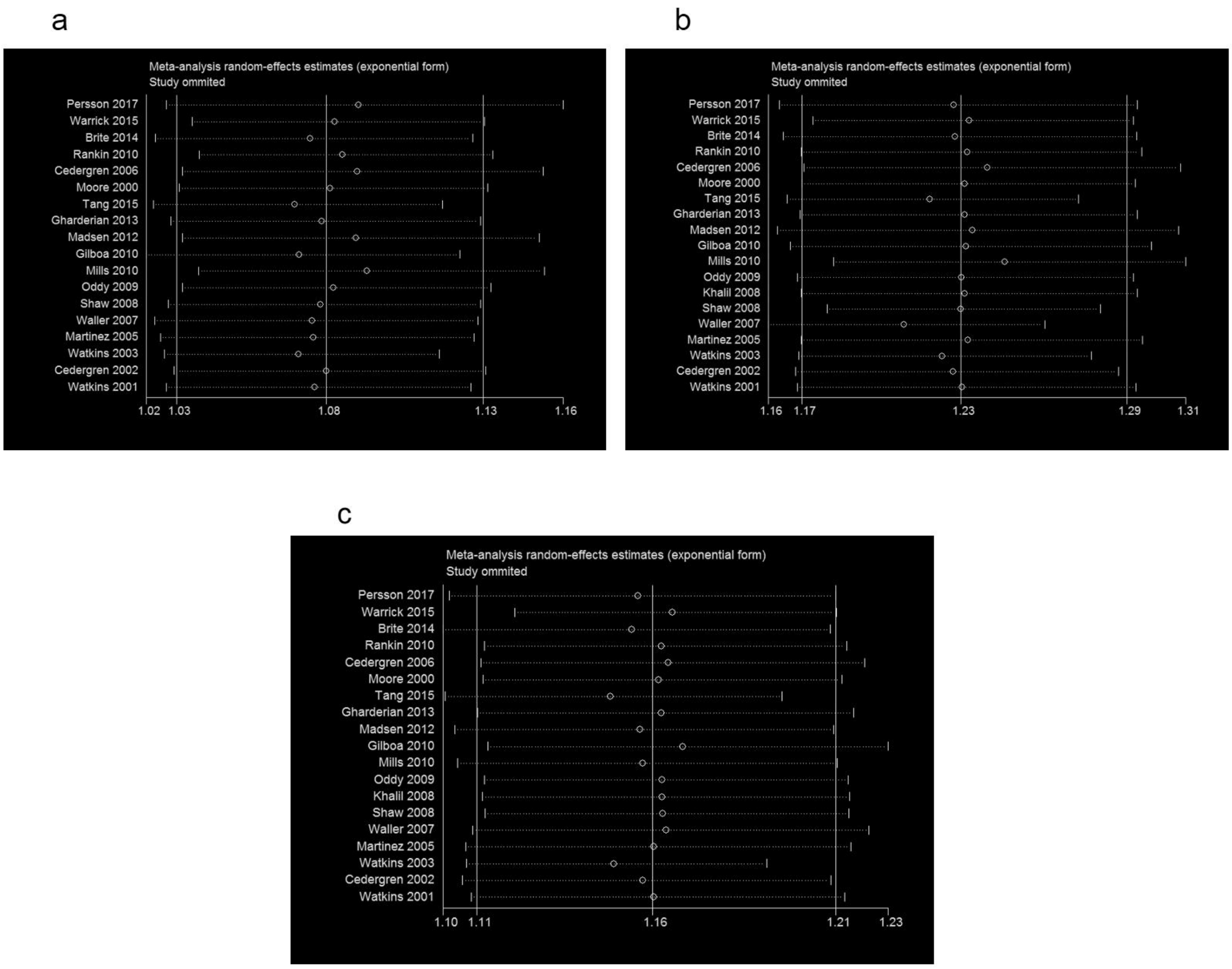

Supplementary Figure 1: Sensitivity analysis corresponding to the random-effects meta-analysis of the relationship between (a) maternal overweight and risk of CHDs in infants; **(b)** maternal obesity and risk of CHDs in infants; **(c)** maternal BMI and risk of CHDs in infants. BMI, body mass index; CHDs, congenital heart defects.

Supplementary Table

Supplementary Table 1. The checklist of PRISMA Statement

Section/Topic	Checklist Item	Reported or not	Reported on page #
TITLE			
Title	Identify the report as a systematic review, meta-analysis, or both.	Y	page 1
ABSTRACT			
Structured summary	Provide a structured summary including, as applicable: background; objectives; data sources; study eligibility criteria, participants, and interventions; study appraisal and synthesis methods; results; limitations; conclusions and implications of key findings; systematic review registration number.	Y	page 2
INTRODUCTION			
Rationale	Describe the rationale for the review in the context of what is already known.	Y	page 2, 3
Objectives	Provide an explicit statement of questions being addressed with reference to participants, interventions, comparisons, outcomes, and study design (PICOS).	Y	page 3
METHODS			
Protocol and registration	Indicate if a review protocol exists, if and where it can be accessed (e.g., Web address), and, if available, provide registration information including registration number.	Y	page 3
Eligibility criteria	Specify study characteristics (e.g., PICOS, length of follow-up) and report characteristics (e.g., years considered, language, publication status) used as criteria for eligibility, giving rationale.	Y	page 3
Information sources	Describe all information sources (e.g., databases with dates of coverage, contact with study authors to identify additional studies) in the search and date last searched.	Y	page 3
Search	Present full electronic search strategy for at least one database, including any limits used, such that it could be repeated.	Y	page 3
Study selection	State the process for selecting studies (i.e., screening, eligibility, included in systematic review, and, if applicable, included in the meta-analysis).	Y	page 3
Data collection process	Describe method of data extraction from reports (e.g., piloted forms, independently, in duplicate) and any processes for obtaining and confirming data from investigators.	Y	page 4
Data items	List and define all variables for which data were sought (e.g., PICOS, funding sources) and any assumptions and simplifications made.	Y	page 4

Risk of bias in individual studies	Describe methods used for assessing risk of bias of individual studies (including specification of whether this was done at the study or outcome level), and how this information is to be used in any data synthesis.	Y	page 4
Summary measures	State the principal summary measures (e.g., risk ratio, difference in means).	Y	page 4
Synthesis of results	Describe the methods of handling data and combining results of studies, if done, including measures of consistency (e.g., I2) for each meta-analysis.	Y	page 4, 5
Risk of bias across studies	Specify any assessment of risk of bias that may affect the cumulative evidence (e.g., publication bias, selective reporting within studies).	Y	page 5
Additional analyses	Describe methods of additional analyses (e.g., sensitivity or subgroup analyses, meta-regression), if done, indicating which were pre-specified.	Y	page 5
RESULTS			
Study selection	Give numbers of studies screened, assessed for eligibility, and included in the review, with reasons for exclusions at each stage, ideally with a flow diagram.	Y	page 5
Study characteristics	For each study, present characteristics for which data were extracted (e.g., study size, PICOS, follow-up period) and provide the citations.	Y	page 5
Risk of bias within studies	Present data on risk of bias of each study and, if available, any outcome-level assessment (see Item 12).	Y	page 5, 6
Results of individual studies	For all outcomes considered (benefits or harms), present, for each study: (a) simple summary data for each intervention group and (b) effect estimates and confidence intervals, ideally with a forest plot.	Y	page 5, 6
Synthesis of results	Present results of each meta-analysis done, including confidence intervals and measures of consistency.	Y	page 5, 6, 7
Risk of bias across studies	Present results of any assessment of risk of bias across studies (see Item 15).	Y	page 7
Additional analysis	Give results of additional analyses, if done (e.g., sensitivity or subgroup analyses, meta-regression [see Item 16]).	Y	page 7
DISCUSSION		Y	
Summary of evidence	Summarize the main findings including the strength of evidence for each main outcome; consider their relevance to key groups (e.g., health care providers, users, and policy makers).	Y	page 7, 8
Limitations	Discuss limitations at study and outcome level (e.g., risk of bias), and at review level (e.g.,	Y	page 8, 9

	incomplete retrieval of identified research, reporting bias).		
Conclusions	Provide a general interpretation of the results in the context of other evidence, and implications for future research.	Y	page 9
FUNDING			
Funding	Describe sources of funding for the systematic review and other support (e.g., supply of data)	Y	page 1

Note: Y, the item was reported in article; N, the item was not reported.
